# Etiology, Risk Factors, and Outcomes of Bacteremia in Patients With Hematologic Malignancies and Febrile Neutropenia in Uganda

**DOI:** 10.1093/ofid/ofae682

**Published:** 2024-11-16

**Authors:** Margaret Lubwama, Sarah E Holte, Yuzheng Zhang, Kelvin R Mubiru, George Katende, Jackson Orem, David P Kateete, Freddie Bwanga, Warren Phipps

**Affiliations:** Department of Medical Microbiology, School of Biomedical Sciences, College of Health Sciences, Makerere University, Kampala, Uganda; Vaccine and Infectious Diseases Division, Fred Hutchinson Cancer Center, Seattle, Washington, USA; Vaccine and Infectious Diseases Division, Fred Hutchinson Cancer Center, Seattle, Washington, USA; Hutchinson Centre Research Institute of Uganda, Kampala, Uganda; Department of Medical Microbiology, School of Biomedical Sciences, College of Health Sciences, Makerere University, Kampala, Uganda; Uganda Cancer Institute, Kampala, Uganda; Department of Immunology and Molecular Biology, School of Biomedical Sciences, College of Health Sciences, Makerere University, Kampala, Uganda; Department of Immunology and Molecular Biology, School of Biomedical Sciences, College of Health Sciences, Makerere University, Kampala, Uganda; Vaccine and Infectious Diseases Division, Fred Hutchinson Cancer Center, Seattle, Washington, USA; Allergy and Infectious Diseases Division, Department of Medicine, University of Washington, Seattle, Washington, USA

**Keywords:** antimicrobial resistance, bacteremia, febrile neutropenia, hematologic malignancies, Uganda

## Abstract

**Background:**

We determined the etiology, risk factors, and outcomes associated with bacteremia in patients with hematologic malignancies and febrile neutropenia (FN) at the Uganda Cancer Institute (UCI).

**Methods:**

UCI adult and pediatric inpatients with hematologic malignancies and FN were prospectively enrolled and followed up to determine 30-day mortality. Blood drawn from participants with FN was cultured in the BACTEC 9120 blood culture system. Antimicrobial susceptibility testing was performed with the disk diffusion method on identified bacteria. Logistic regression and Cox proportional hazards regression were applied to estimate associations between participant characteristics and FN, bacteremia, and mortality.

**Results:**

Of 495 participants, the majority (n = 306 [62%]) were male. Median age was 23 years (interquartile range, 11–42 years). Of the 132 participants who experienced FN, 43 (33%) had bacteremia. Participants with younger age (odds ratio [OR], 0.98; *P* = .05), severe neutropenia (OR, 2.9; *P* = .01), hypotension (OR, 2.46; *P* = .04), mucositis (OR, 2.77; *P* = .01), and receipt of chemotherapy (OR, 2.25; *P* = .03) were more likely to have bacteremia. Fifty (78%) bacteria isolated were gram negative. *Escherichia coli* (n = 25 [50%]) was predominant. Thirty-seven of 43 (86%) episodes were caused by multidrug-resistant (MDR) bacteria. Thirty-day overall survival for participants with bacteremia was significantly lower than that for participants with no bacteremia (*P* = .05). MDR bacteremia (hazard ratio, 1.84; *P* = .05) was associated with increased risk of death.

**Conclusions:**

Bacteremia was frequent in patients with hematologic cancer and FN and was associated with poor survival. MDR bacteria were the main cause of bacteremia and mortality. There is a need for robust infection control and antimicrobial stewardship programs in cancer centers in sub-Saharan Africa.

Cancer is a growing public health problem in sub-Saharan Africa (SSA), where mortality rates are significantly higher than in high-income countries (HICs) [1]. While several factors contribute to poor cancer outcomes in SSA, infectious complications of cancer are likely an important contributor to high mortality. Among patients with cancer and febrile neutropenia (FN), bacteremia is a particularly life-threatening complication [2], and effective management depends on prompt administration of appropriate empiric therapy as guided by the epidemiology of organisms in the healthcare environment and the broader community [3, 4].

Despite the critical need for reliable local data to guide management of FN, little is known about the causes, risk factors, or outcomes of bloodstream infections among patients with cancer in SSA [4]. Moreover, unique epidemiologic factors in Africa, including the high prevalence of human immunodeficiency virus (HIV) infection and the growing threat of antimicrobial resistance (AMR), shape the local spectrum of bacterial diseases that make extrapolation from management guidelines in other regions of the world unreliable.

Among the few studies that have evaluated infections in patients with cancer in SSA, bacteremia was common, ranging from 7% to 35% of cultures [5–12]. Several of these studies reported AMR among the isolates, including methicillin-resistant *Staphylococcus aureus* (MRSA) and presence of extended-spectrum β-lactamases (ESBLs) [7, 8, 11, 12]. In our previous study of bacteremia among patients at the Uganda Cancer Institute (UCI) in Kampala, Uganda, we found that nearly 85% of Enterobacteriaceae isolated were multidrug resistant (MDR) and that patients with hematologic malignancies had the highest risk of MDR bacteremia [7]. The high rates of AMR are a particular challenge to managing infections in patients with cancer and limit the therapeutic options available for choosing appropriate antimicrobial therapy [13, 14]. While these few studies highlight the importance of MDR bacteremia among patients with cancer in SSA, the studies are generally of small sample size and do not evaluate the risk factors and outcomes of bacteremia that are important for guiding the development of effective interventions. To help address these gaps, we undertook a prospective study to comprehensively characterize the etiology of bacteremia and to identify risk factors and outcomes associated with FN and bacteremia among adult and pediatric inpatients with hematologic malignancies at the UCI.

## METHODS

### Study Design and Setting

We conducted a prospective cohort study from November 2017 to March 2020 of inpatients with hematologic malignancies at the UCI, the national cancer referral center in Kampala, Uganda.

### Study Procedures

All pediatric (aged < or = 15 years) and adult patients (aged >15 years) admitted to the UCI with a hematologic malignancy were offered the opportunity to participate in the study. Hematologic malignancies included acute lymphocytic leukemia (ALL), acute myeloid leukemia (AML), chronic lymphocytic leukemia, chronic myeloid leukemia, Hodgkin lymphoma, non-Hodgkin lymphoma, and myelodysplastic syndrome (MDS). For participants who consented, demographic and clinical data were abstracted from the medical record. Participants and families were provided with a thermometer and temperature logbooks and trained to monitor participant axillary temperatures at least 3 times per day and whenever there was a concern for fever to augment standard-of-care vital sign monitoring by nurses on the ward. Peripheral blood cultures were drawn on all participants who developed FN, defined as an axillary temperature of ≥37.5°C and an absolute neutrophil count (ANC) of ≤1000 cells/µL. We used a lower threshold for temperature (37.5°C) because we were measuring axillary temperature and not oral temperature [15]. HIV testing was provided for participants who developed FN. Blood culture and sensitivity results were provided to UCI physicians to guide antimicrobial selection and management.

### Microbiological Studies

Blood cultures were processed at the College of American Pathologists–accredited Makerere University Clinical Microbiology Laboratory (MUCML). Blood was collected in 2 BACTEC aerobic blood culture bottles (Becton Dickinson) aseptically from 2 peripheral sites from patients with FN. In adult patients, 5–10 mL of blood per bottle was collected, while 3 mL of blood per bottle was collected from pediatric patients.

At the MUCML, the blood samples were processed in the BACTEC 9120 blood culture system (Becton Dickinson). Organisms in positive blood culture samples were identified using Gram stain and culture characteristics on chocolate blood, blood, and MacConkey agars. The agar plates were incubated for 18–24 hours at 35°C–37°C. Conventional biochemical tests were used to definitively identify different bacterial colonies [16]. For organisms that could not be identified using conventional methods, the Phoenix automated system (Becton Dickinson) was used, according to the manufacturer's instructions. Polymicrobial bloodstream infection (PBSI) was defined as bacteremia with >1 pathogen isolated from blood. Coagulase-negative staphylococci (CoNS), *Micrococcus* species, *Corynebacterium* species, and *Bacillus* species were considered potential skin contaminants if they grew from 1 bottle only and were not included in the analyses.

Antimicrobial susceptibility testing was performed using the Kirby-Bauer disk diffusion method for the different bacteria following the laboratory's standard operating procedures and Clinical and Laboratory Standard Institute (CLSI) guidelines [17]. Multidrug resistance was defined as an isolate being nonsusceptible to at least 1 agent in ≥3 antimicrobial categories [18]. The screening of MDR phenotypes including ESBL-producing Enterobacterales using the double disk synergy test method, MRSA, and ampicillin- and vancomycin-resistant enterococci were carried out following the CLSI guidelines. Control strains (*Escherichia coli* ATCC 25922, *Pseudomonas aeruginosa* ATCC 27853, *Klebsiella pneumoniae* ATCC 700603, and *S aureus* ATCC 25923) were used for quality control.

### Statistical Analysis

Febrile neutropenia was defined as an axillary temperature of ≥37.5°C and an ANC of ≤1000 cells/µL. Severe neutropenia was defined as neutrophil count of <100 cells/µL. Chemotherapy at time of FN was defined as chemotherapy received by patients within 1 month before FN onset. Previous antibiotic therapy was defined as antibiotic therapy received by the patient within the past 3 months before the FN episode as recorded in the medical records. Previous hospitalization was defined as hospitalization within the past 3 months before FN onset. Treatment guidelines at the UCI included piperacillin-tazobactam and gentamicin as first-line treatment and meropenem as second-line treatment [19]. Empirical antibiotic therapy was defined as documented antibiotics administered within 2 days of FN. Inappropriate empiric antibiotic therapy was defined as a documented treatment regimen that did not include at least 1 antibiotic that was active in vitro against the isolated organism. Overall case-fatality rate was defined as death by any cause within 30 days of onset of the first FN episode.

Descriptive data were reported as frequencies or as median (interquartile range [IQR]). To test for the difference in distributions, we conducted Mann-Whitney *U* tests for continuous variables. Categorical variables were evaluated with the χ^2^ test. Logistic regression was conducted to evaluate factors associated with developing FN and with bacteremia among those with FN. Odds ratio (ORs), 95% confidence intervals (CIs), and *P* values were reported. To investigate the factors associated with 30-day overall mortality in patients from the first FN, Cox proportional hazards regression was conducted using the 30-day follow-up time since the FN onset. Hazard ratios (HRs), 95% CIs, and *P* values were reported. Kaplan-Meier curves and log-rank tests were used to compare the survival probability between bacteremia versus nonbacteremia for the 30 days after first FN onset. A *P* value of ≤.05 (2-tailed) was considered to be statistically significant. Analysis was carried out using R version 4.3.1 software (R Foundation for Statistical Computing, Vienna, Austria).

### Ethics

We received ethical approval from the Institutional Review Board (IRB) of the School of Biomedical Sciences, College of Health Sciences, Makerere University (SBS-396), the Uganda National Council of Science and Technology (HS 2217), and the Fred Hutchinson Cancer Research Center IRB (FHCC #8433). Written informed consent was obtained from all participants. Assent was obtained from children between 8 and 17 years in addition to parental informed consent. If the patient was too ill to provide informed consent for the study, consent was sought from the patient's next of kin.

## RESULTS

### Characteristics of Study Participants and Factors Associated With Developing FN

A total of 495 participants were enrolled in the study. Median age of the participants was 23 years (IQR, 11–42 years) and most (n = 306 [62%]) were male (Table 1). One hundred thirty-two of 495 (27%) participants developed FN (Supplementary Figure 1). In univariate analysis, factors associated with FN included younger age (OR, 0.98 per year older; *P* < .01), HIV-negative serostatus (OR, 5.36; *P* < .01), and previous hospitalization (OR, 1.58; *P* = .03). Among types of hematologic malignancies, higher proportions of patients who developed FN included AML (52/94 [55%]), ALL (42/106 [40%]), and MDS (6/19 [32%]). The association between the development of FN with cancer type was attenuated but remained in univariate analysis adjusted for HIV status and adult/child variables (Supplementary Table 1).

**Table 1.** Patient Characteristics by Febrile Neutropenia Episode Status

Characteristic	Overall	FNE	No FNE	OR (95% CI)	*P* Value
	No.	No. (%)	No. (%)		
Total	495	132 (27)	363 (73)		
Age, y, median (IQR)	23 (11–42)	19 (10–26)	25 (11–47)	0.98 (.95–.99)	<.01
Ward					
Adult	315	83 (26)	232 (74)	Ref	
Pediatric	180	49 (27)	131 (73)	1.05 (.69–1.58)	.83
Sex					
Female	189	49 (26)	140 (74)	Ref	
Male	306	83 (27)	223 (73)	1.06 (.71–1.61)	.77
Cancer diagnosis					
ALL	106	42 (40)	64 (60)	Ref	
AML	94	52 (55)	42 (45)	1.89 (1.08–3.33)	.03
CLL	10	1 (10)	9 (90)	0.17 (.02–1.39)	.10
CML	33	5 (15)	28 (85)	0.27 (.09–.71)	.01
HL	27	3 (11)	24 (89)	0.19 (.04–.59)	.01
NHL	166	22 (13)	144 (87)	0.23 (.13–.42)	<.001
Multiple myeloma	39	1 (3)	38 (97)	0.04 (0–.2)	<.01
MDS	19	6 (32)	13 (68)	0.7 (.23–1.93)	.51
Other	1	0 (0)	1 (100)	…	
HIV status					
Positive	55	5 (9)	50 (91)	Ref	
Negative	318	111 (35)	207 (65)	5.36 (2.28–15.76)	.001
Unknown	122	16 (13)	106 (87)	1.51 (.56–4.82)	.446
Previous hospitalization					
No	216	47 (22)	169 (78)	Ref	
Yes	279	85 (30)	194 (70)	1.58 (1.05–2.39)	.03

Abbreviations: ALL, acute lymphocytic leukemia; AML, acute myeloid leukemia; CI, confidence interval; CLL, chronic lymphocytic leukemia; CML, chronic myeloid leukemia; FNE, febrile neutropenia episode; HIV, human immunodeficiency virus; HL, Hodgkin lymphoma; IQR, interquartile range; NHL, non-Hodgkin lymphoma; MDS, myelodysplastic syndrome; OR, odds ratio.

### Factors Associated With Bacteremia Among Participants With FN

Of the 132 participants who developed FN, 43 (33%) had bacteremia (Table 2). In univariate analysis, younger patients were more likely than older patients to have bacteremia (OR, 0.98 per year older; *P* = .05). ANC <100 cells/μL (OR, 2.9; *P* = .01), hypotension (OR, 2.46; *P* = .04), and mucositis (OR, 2.77; *P* = .01) were also associated with bacteremia. Participants receiving chemotherapy were more likely than those not on treatment to have bacteremia (OR, 2.25; *P* = .03). HIV serostatus was not associated with bacteremia. In the multivariate model “Logit(bacteremia/no bacteremia) ∼ Age + Chemo at FN episode + ANC <100 + Hypotension + Mucositis,” the associations were attenuated and no longer remained significant (Supplementary Table 2).

**Table 2.** Epidemiological and Clinical Characteristics of Patients by Bacteremia Status

Patient Characteristics	Overall, No.	Bacteremia	No Bacteremia	OR (95% CI)	*P* Value
		No. (%)	No. (%)		
Total	132	43	89		
Age, y, median (IQR)	19	16 (10–26)	20 (10–34)	0.98 (.95–1)	.05
Ward					
Adult	83	25 (30)	58 (70)	Ref	
Pediatric	49	18 (37)	31 (63)	1.35 (.63–2.84)	.43
Sex					
Female	49	19 (39)	30 (61)	Ref	
Male	83	24 (29)	59 (71)	0.64 (.3–1.36)	.24
Cancer diagnosis					
ALL	42	14 (33)	28 (67)	Ref	
AML	52	21 (40)	31 (60)	1.35 (.58–3.2)	.48
CLL	1	0	1 (100)	…	
CML	5	1 (20)	4 (80)	0.5 (.02–3.79)	.55
HL	3	1 (33)	2 (67)	…	
NHL	22	4 (18)	18 (82)	0.44 (.11–1.47)	.21
Multiple myeloma	1	0	1 (100)	…	
MDS	6	2 (33)	4 (67)	1 (.13–5.8)	1
HIV status					
Negative	111	37 (33)	74 (67)	Ref	
Positive	5	1 (20)	4 (80)	0.5 (.03–3.53)	.54
Unknown	16	5 (31)	11 (69)	0.91 (.27–2.7)	.87
Previous hospitalization					
No	47	16 (34)	31 (66)	Ref	
Yes	85	27 (32)	58 (68)	0.9 (.43–1.94)	.79
Previous antibiotics					
No	18	4 (22)	14 (78)	Ref	
Yes	114	39 (34)	75 (66)	1.73 (.5–8.06)	.42
Patient on chemotherapy at FNE					
No	70	17 (24)	53 (76)	Ref	
Yes	62	26 (42)	36 (58)	2.25 (1.08–4.8)	.03
ANC <100 cells/μL					
No	81	19 (23)	62 (77)	Ref	
Yes	51	24 (47)	27 (53)	2.9 (1.38–6.23)	.01
Hypotension					
No	104	29 (28)	75 (72)	Ref	
Yes	28	14 (50)	14 (50)	2.46 (1.03–5.93)	.04
Anemia					
No	76	24	52	Ref	
Yes	56	19	37	1.11 (.53–2.32)	.78
Thrombocytopenia					
No	6	0	6 (100)	Ref	
Yes	126	43 (34)	83 (66)	…	
Mucositis					
No	98	26 (27)	72 (73)	Ref	
Yes	34	17 (50)	17 (50)	2.77 (1.23–6.27)	.01

Abbreviations: ALL, acute lymphocytic leukemia; AML, acute myeloid leukemia; ANC, absolute neutrophil count; CI, confidence interval; CLL, chronic lymphocytic leukemia; CML, chronic myeloid leukemia; FNE, febrile neutropenia episode; HIV, human immunodeficiency virus; HL, Hodgkin lymphoma; IQR, interquartile range; NHL, non-Hodgkin lymphoma; MDS, myelodysplastic syndrome; OR, odds ratio.

### Microbial Etiology of FN

**Figure 1.**
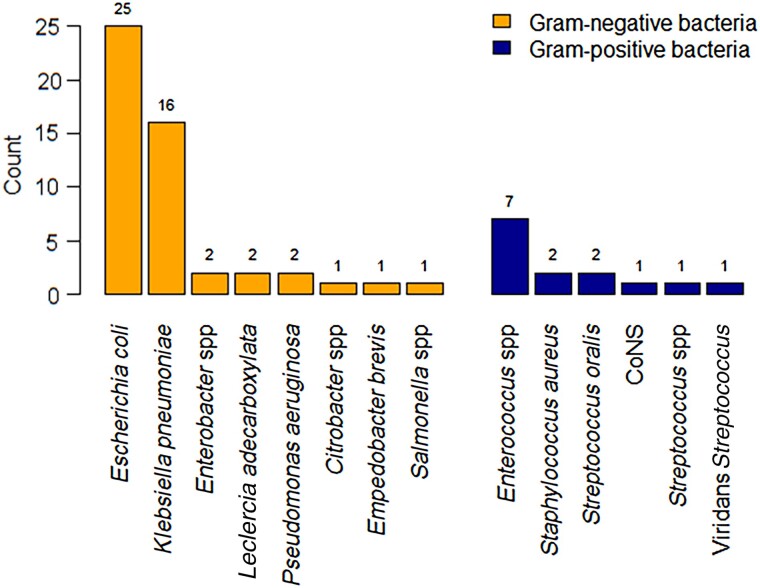
Bacteria isolated from patients with hematologic cancer and febrile neutropenia. Abbreviation: CoNS, coagulase-negative staphylococci.

Of 132 FN episodes, 43 had true pathogens (bacteria), 20 had contaminants, and 69 were negative. The contaminants included CoNS (11), *Micrococcus* species (7), and *Bacillus* species (2). Of the 64 pathogenic bacterial isolates from the 43 bacteremia cases, 50 (78%) were gram negative (Figure 1). The bacteria isolated in the different populations (adults/pediatric) have been included in Supplementary Table 3. The most common gram-negative bacteria were the Enterobacterales, of which *E coli* (n = 25 [50%]) and *K pneumoniae* (n = 16 [32%]) were dominant. Less common gram-negative isolates included *Enterobacter* species (n = 2), *Citrobacter* species (n = 1), *Salmonella* (n = 1), *Leclercia adecarboxylata* (n = 2), *P aeruginosa* (n = 2), and *Empedobacter brevis* (n = 1). The most common gram-positive bacteria were *Enterococcus* species (n = 7 [50%]). Other gram-positive isolates included *S aureus* (n = 2), *Streptococcus* species (n = 4), and CoNS (n = 1).

Fifteen of the 43 (35%) bacteremia episodes were PBSI (Supplementary Table 4). All but 1 PBSI included at least 1 gram-negative organism. Eight of the 15 (53%) PBSIs included only gram-negative bacteria, 4 (27%) included both gram-negative and gram-positive bacteria, and 2 (13%) included gram-negative bacteria and fungi (mold). One (7%) PBSI included only gram-positive bacteria. Nine of the 15 (60%) PBSIs had 2 organisms, 4 (27%) had 3 organisms, and 2 (13%) had 4 organisms. In cases of PBSI, *E coli* (17/25 [68%]) was the most frequently isolated gram-negative while *Enterococcus* species (4/7 [57%]) was the most frequently isolated gram-positive.

### Antimicrobial Susceptibility Testing of Bacterial Isolates

The antibiogram for bacteria isolated is shown in Table 3 (against β-lactam antibiotics) and Table 4 (against antibiotics other than β-lactams). Overall, 37 of the 43 (86%) bacteremia episodes were caused by at least 1 MDR organism.

**Table 3.** Antibiogram for Bacteria Showing Percentage Susceptible (β-Lactam Antibiotics)

Organism	Ampicillin	Penicillin	Piperacillin	Amox-Clav	Amp-Sulb	Pip-Tazo	Cefazolin	Cefuroxime	Cefotaxime	Ceftriaxone	Ceftazidime	Cefepime	Cefoxitin	Cefotetan	Aztreonam	Ertapenem	Imipenem	Meropenem
*Escherichia coli* (n = 25)	0^f^	–	–	16^f^	0^a,f^	16^f^	4^a,f^	8^a,f^	12^f^	12^f^	12^f^	16^f^	64^e^	62^a,e^	12^f^	56^f^	72^e^	72^e^
*Klebsiella pneumoniae* (n = 16)	0^f^	–	–	19^f^	13^f^	31^f^	7^b,f^	12^f^	7^b,f^	12^f^	25^f^	7^b,f^	100^b,d^	100^b,d^	13^b,f^	87^b,d^	94^d^	100^b,d^
*Enterobacter* spp (n = 2)	0^f^	–	–	50^f^	50^f^	100^d^	50^f^	100^d^	100^d^	100^d^	100^d^	100^d^	100^d^	100^d^	100^d^	100^d^	100^d^	100^d^
*Citrobacter* spp (n = 1)	0^f^	–	–	0^f^	0^f^	0^f^	0^f^	0^f^	0^f^	0^f^	0^f^	0^f^	100^d^	100^d^	0^f^	0^f^	100^d^	100^d^
*Salmonella* (n = 1)	100^d^	–	–	100^d^	0	100^d^	100^d^	100^d^	100^d^	100^d^	100^d^	100^d^	100^d^	100^d^	100^d^	100^d^	100^d^	100^d^
*Leclercia adecarboxylata* (n = 2)	0^f^	–	–	0^f^	0^f^	0^f^	0^f^	100^d^	100^d^	100^d^	50^f^	100^d^	0^f^	50^f^	100^d^	100^d^	100^d^	100^d^
*Pseudomonas aeruginosa* (n = 2)	–	–	100^d^	–	–	100^d^	–	–	–	–	100^d^	100^d^	–	–	–	100^d^	100^d^	100^d^
*Empedobacter brevis* (n = 1)	0^f^	–	–	0^f^	0^f^	0^f^	0^f^	0^f^	0^f^	0^f^	0^f^	0^f^	–	100^d^	100^d^	0^f^	0^f^	0^f^
*Enterococcus* spp (n = 7)	0	0	–	–	–	–	–	–	–	–	–	–	–	–	–	–	–	–
Other *Streptococcus* spp (n = 4)	25^c,f^	–	–	–	–	–	–	–	–	0^f^	–	–	–	–	–	–	–	–
*Staphylococcus aureus* (n = 2)	–	0^f^	–	–	–	–	–	–	–	–	–	–	50^f^	–	–	–	–	–
CoNS (n = 1)	–	0^f^	–	–	–	–	–	–	–	–	–	–	100^d^	–	–	–	–	–

Abbreviations: –, not tested; Amox-Clav, amoxicillin-clavulanate; Amp-Sulb, ampicillin-sulbactam; CoNS, coagulase-negative staphylococci; Pip-Tazo, piperacillin-tazobactam.

^a^Twenty-four organisms tested.

^b^Fifteen organisms tested.

^c^One organism tested.

^d^>80% of isolates susceptible.

^e^60%–80% of isolates susceptible.

^f^<60% of isolates susceptible.

**Table 4.** Antibiogram for Bacteria Showing Percentage Susceptible (Drugs Other Than β-Lactams)

Organism	Aminoglycosides		Fluoroquinolones	Folate Pathway Inhibitors	Phenicols	Glycopeptide	Macrolide	
	Gentamicin	Amikacin	Ciprofloxacin	SXT	Chloramphenicol	Vancomycin	Erythromycin	Clindamycin
*Escherichia coli* (n = 25)	40^c^	52^c^	8^c^	0^c^	72^b^	–	–	–
*Klebsiella pneumoniae* (n = 16)	25^c^	80^b^	20^c^	7^c^	53^c^	–	–	–
*Enterobacter* spp (n = 2)	100^a^	100^a^	50^c^	50^c^	100^a^	–	–	–
*Citrobacter* spp (n = 1)	0^c^	100^a^	0^c^	0^c^	0^c^	–	–	–
*Salmonella* (n = 1)	100^a^	100^a^	100^a^	100^a^	100^a^	–	–	–
*Pseudomonas aeruginosa* (n = 2)	100^a^	100^a^	100^a^	–	–	–	–	–
*Leclercia adecarboxylata* (n = 2)	100^a^	100^a^	0^c^	100^a^	100^a^	–	–	–
*Empedobacter brevis* (n = 1)	–	100^a^	0^c^	0^c^	100^a^	–	–	–
*Enterococcus* spp (n = 7)	57^c^	–	0^c^	–	43^c^	100^a^	0^c^	–
Other *Streptococcus* spp (n = 4)	–	–	0^c^	–	75^b^	75^b^	25^c^	75^b^
*Staphylococcus aureus* (n = 2)	100^a^	–	50^c^	50^c^	100^a^	100^a^	50^c^	100^a^
CoNS (n = 1)	100^a^	–	0^c^	0^c^	100^a^	100^a^	100^a^	100^a^

Abbreviations: –, not tested; CoNS, coagulase-negative staphylococci; SXT, Sulfamethoxazole-trimethoprim.

^a^>80% of isolates susceptible.

^b^60%–80% of isolates susceptible.

^c^<60% of isolates susceptible.

Of 47 Enterobacterales, 38 (81%) were nonsusceptible to third-generation cephalosporins, and 29 of these 38 (76%) Enterobacterales were positive for the ESBL phenotype. Among the 47 Enterobacterales isolates, 32 (68%) were susceptible to ertapenem, 39 (83%) were susceptible to imipenem, and 39 (83%) were susceptible to meropenem. Among the 3 non–lactose fermenters, 2 *P aeruginosa* isolates were susceptible to all antibiotics. The *E brevis* isolated was MDR.

Among gram-positive bacteria, all *Enterococcus* species were MDR. All were ampicillin resistant and vancomycin sensitive. Three *Enterococcus* species (43%) were resistant to high-level gentamicin. One *Streptococcus* species (25%) was vancomycin resistant. One (50%) *S aureus* was methicillin resistant. The only CoNS isolated was methicillin sensitive.

### Antibiotic Use

The most common documented antibiotics prescribed at FN onset were piperacillin-tazobactam and gentamicin (Supplementary Figure 2). Only 8 (19%) participants with bacteremia received appropriate antibiotics within 2 days of the onset of FN. By 7 days of the FN, 15 (35%) participants with bacteremia had received appropriate antibiotics. A higher proportion of participants with inappropriate antibiotic use had MDR bacteremia compared to participants with inappropriate antibiotic use and no MDR bacteremia (71% vs 6%, *P* < .01).

### Survival and Factors Associated With Mortality

**Figure 2.**
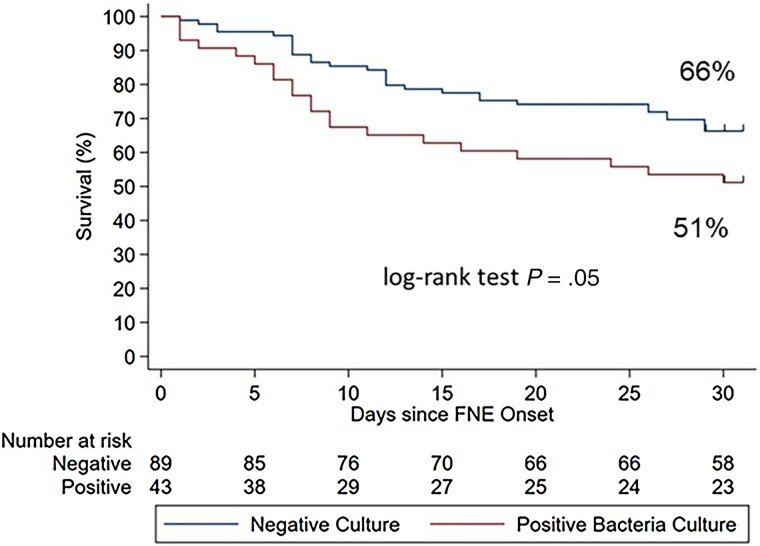
Thirty-day overall survival for patients with hematologic cancer and febrile neutropenia by culture status. Abbreviation: FNE, febrile neutropenia episode.

At 30 days following onset of FN, 51 (38%) participants had died. The 30-day overall survival for participants with bacteremia was significantly lower than that of participants with no bacteremia (*P* = .05) (Figure 2), with a 30-day overall survival of only 51% among FN participants with bacteremia compared to 66% without bacteremia.

**Figure 3.**
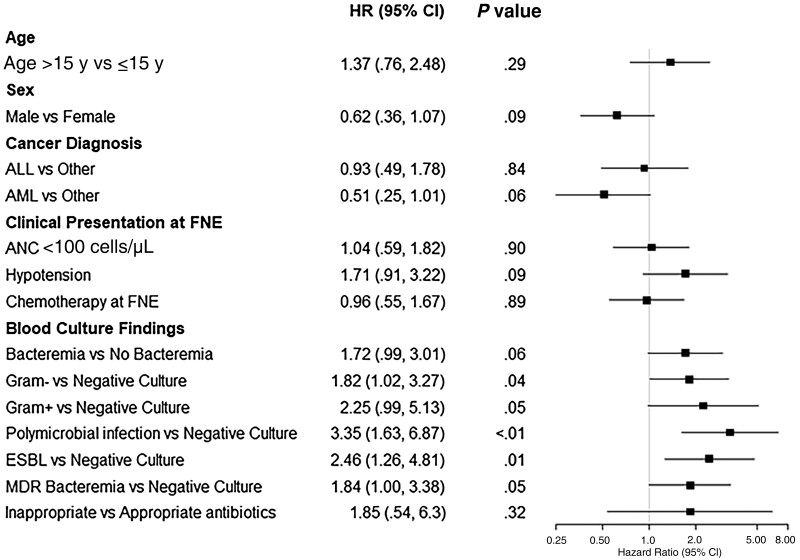
Univariate analyses of factors associated with 30-day mortality in patients with hematologic cancer and febrile neutropenia. Abbreviations: ALL, acute lymphocytic leukemia; AML, acute myeloid leukemia; ANC, absolute neutrophil count; CI, confidence interval; ESBL, extended-spectrum β-lactamase; FNE, febrile neutropenia episode; HR, hazard ratio; MDR, multidrug resistant.

Characteristics of patients with hematologic cancers and bacteremia who were dead at 30 days from FN are shown in Supplementary Table 5. In univariate analysis (Figure 3), factors associated with increased risk of death included gram-negative bacteremia (HR, 1.82 [95% CI, 1.02–3.27]; *P* = .04), gram-positive bacteremia (HR, 2.25 [95% CI, .99–5.13]; *P* = .05), PBSI (HR, 3.35 [95% CI, 1.63–6.87]; *P* < .01), ESBL-positive, gram-negative bacteremia (HR, 2.46 [95% CI, 1.26–4.81]; *P* = .01), and MDR bacteremia (HR, 1.84 [95% CI, 1–3.38]; *P* = .05) compared to negative culture. HIV infection was not associated with mortality among participants with FN.

## DISCUSSION

To our knowledge, this is the first prospective study conducted in SSA to describe the etiology of bacteremia and to assess the associated risk factors and survival outcomes in patients with hematologic cancer and FN. Almost one-third (27%) of the patients developed FN, which is higher than what has been reported in similar studies in HICs [14]. We found the highest proportion of FN in patients with AML and ALL, which is consistent with previous observations that patients with acute leukemias are at high risk of developing infections, due to functional neutropenia associated with these cancers and intensive chemotherapy regimens used for treatment [3]. Patients with previous hospitalization were more likely to have FN. Previously hospitalized patients have had exposure to pathogens in the hospital environment, predisposing them to colonization and subsequent infections, a major cause of FN [20].

Of note, we found that patients with hematologic cancer and HIV were less likely to have FN than patients with cancer and without HIV. Our finding may be due in part to the observation that lymphomas are more common among people with HIV, whereas most of the patients with FN in our cohort had leukemia [21]. Additionally, persons with HIV may also receive more prophylactic antibiotics as part of their HIV care or be less likely to mount a detectable fever if they are severely immunocompromised [22]. Future research on how HIV infection influences FN presentation and outcomes is warranted.

We observed a high rate (33%) of bacteremia in our study. Other studies of patients with cancer in SSA have reported bacteremia ranging from 7% to 35%, including a prior study from our institute that identified bacteremia in 14% of febrile patients with cancer [5, 7–12]. The higher rate of bacteremia we observed is most likely because our study focused on the high-risk category of hematologic malignancies and FN. Increased availability of chemotherapy over time may have led to the use of more toxic treatment regimens, which may also have increased the likelihood of infection in our cohort. Nonetheless, the high rate of bacteremia we observed highlights that bacterial infection remains a common and important problem among patients in Africa with hematologic malignancies, particularly those with leukemia.

We found several factors that were associated with bacteremia, including severe neutropenia, mucositis, hypotension, and recent chemotherapy. Cytotoxic chemotherapy causes prolonged, severe neutropenia and contributes to mucositis, which creates an entry point for resident bacteria to cross into the bloodstream [23]. Of note, HIV serostatus was not associated with bacteremia in our study. Other infections more common in people with HIV, including tuberculosis and disseminated mycosis, may contribute to FN and need further study [24].

Gram-negative bacteria were the predominant cause of bacteremia, with *E coli* being the most frequent bacteria isolated. HICs have demonstrated a shift over time from predominantly gram-negative to gram-positive infections, yet most studies in low- and middle-income countries (LMICs) have demonstrated that gram-negative bacteria remain the most common cause of bloodstream infections [7, 25–27]. This difference could be due to the variations in clinical practices that predispose to gram-positive bacteremia in HICs, including more frequent use of central venous catheters (CVCs), which favor colonization of gram-positive bacteria, and use of fluoroquinolone prophylaxis, which targets gram-negative bacteria [25, 28]. While fluoroquinolone prophylaxis is given to patients with acute leukemias, none of the patients at the UCI at the time of the study had CVCs. However, currently, AMR is common in both gram-positive and gram-negative bacteria isolated from cancer centers in both HICs and LMICs [29, 30].

Notably, we found that more than one-third of episodes of bacteremia were PBSI, with all PBSI having at least 1 gram-negative isolate. Consistent with previous observations, *E coli* and *Enterococcus* species were the most predominant bacteria, with a higher proportion of PBSI involving at least 1 gram-negative isolate [31, 32]. The high frequency of PBSI with gram-negative bacteria likely reflects seeding from the gastrointestinal tract in the setting of gastrointestinal mucositis [33].

Alarmingly, most (86%) of the bacteremia episodes in our study were caused by at least 1 MDR bacterium. Notably, most patients in the study, both with and without bacteremia, had a history of previous antibiotic use. Prophylactic antibiotics (levofloxacin) are given to patients with cancer, especially those with acute leukemia. While there was no difference between the bacteremia and nonbacteremia groups, we observed that a significantly higher proportion of patients with FN reported previous hospitalization within 3 months, hence more frequent healthcare exposure. Previous antibiotic and hospital exposure are risk factors for the development of resistance [34].

The proportion of ESBL phenotype demonstrated in this study was higher than the 41% that we previously reported in the same institution [7]. Other cancer institutes have reported increased proportions of ESBL-positive Enterobacterales isolated from their patients with bacteremia [35, 36]. More than one-quarter of our *E coli* isolates were resistant to meropenem and imipenem. Carbapenem resistance has also been reported in cancer centers in HICs [37]. Carbapenem-resistant bacteria present a major concern in the management of FN patients because they limit the therapeutic options for severe infections, especially in the LMIC setting [30, 38]. While the most common mechanism of carbapenem resistance is due to the presence of carbapenemases, we observed differences in resistance between ertapenem and imipenem-meropenem, which could be explained by presence of CTX-M ESBL plus porin loss. We have reported a high proportion of CTX-M in the Enterobacterales we isolated from this patient population [39]. The *Enterococcus* species isolated in this study were all resistant to ampicillin, and almost half were resistant to high-level gentamicin. The presence of high-level aminoglycoside resistance negates ampicillin-aminoglycoside synergism [40]. Given the high rates of MDR bacteria observed, further study and monitoring of the transmission and acquisition of AMR at cancer centers in SSA are urgently needed.

We observed a 30-day mortality rate of 38% of patients with FN. The overall mortality for FN patients with bacteremia was higher (49%) than that of FN patients with no bacteremia (34%). We showed higher mortality rates than most other studies, which ranged from 25% to 47% [6, 41–45]. This high mortality rate may be in part due to the fact that our study focused on only hematologic malignancies in both adult and pediatric populations, whereas other studies focused on both hematologic and solid tumor malignancies or on only solid malignancies, which may have a lower mortality rate.

Best practice for selecting the appropriate empiric antibiotic to be used for sepsis depends on the epidemiology of organisms in the institution [13]. In our study, only 19% of patients with bacteremia received appropriate empiric antimicrobial therapy within 48 hours of fever onset. Piperacillin-tazobactam and gentamicin were the most common antibiotics prescribed at time of FN, based on our local guidelines [19]. Unfortunately, our study revealed very high resistance against these antibiotics. Given the high rates of MDR bacteria we observed, our institutional guidelines for empiric therapy need to be updated to a broader spectrum, which can then be de-escalated to a narrower spectrum to comport with the antimicrobial susceptibility test result [46]. The drug of choice for ESBL-positive Enterobacterales is a carbapenem, yet nearly a quarter of our isolates also demonstrated a carbapenem-resistant phenotype, making a universal approach to empiric therapy very challenging. Other treatment options for carbapenem-resistant bacteria, such as ceftazidime-avibactam, meropenem-vaborbactam, and cefiderocol, are prohibitively expensive in Uganda and are not available at UCI [47]. Colistin, an accessible option, has been associated with nephrotoxicity and resistance [48]. Similar to other studies in Uganda, we did not observe vancomycin resistance in our gram-positive isolates [49]. This is unlike other cancer centers, which have reported high rates of vancomycin-resistant *Enterococcus* species [50]. Therefore, vancomycin remains the drug of choice for *Enterococcus* species in our center. Notably, MDR bacteria and PBSI, irrespective of the category of organisms, are likely to contribute to the high mortality rates observed, most likely due to their association with inappropriate antimicrobial therapy [32, 35].

AMR has played a significant role in increased mortality in patients with cancer [30]. In our study, patients with MDR bacteremia had a higher risk of 30-day mortality compared to patients with bacteremia caused by susceptible organisms. This is consistent with reports from other studies [51]. Mortality rates in cancer centers in LMICs are higher than those reported in HICs, most likely due to the higher rates of AMR observed in LMICs driven by inappropriate use of antibiotics [52]. In our study, patients who received inappropriate antimicrobial therapy had a higher mortality rate compared to patients who received appropriate antimicrobial therapy, although the difference in risk was not significant. Limited access to appropriate antibiotics contributes to the disproportionate burden of AMR faced in LMICs [34]. Strategies to identify patients with the highest risk for MDR bacteremia and to de-escalate broad empiric therapy are important for effectively utilizing limited antimicrobial resources in LMIC settings and are important areas of future research.

This was a prospective study, which enabled us to determine the etiology, risk factors, and outcomes for bacteremia in patients with hematologic cancer in our institution. It was limited to a single institution and the conclusions may be different from other institutions in different geographical regions. It focused on inpatient hematologic malignancies. The frequency and causes of bacteremia may differ in different patient populations. Additionally, due to the heterogeneity of the hematologic cancer populations, the sample sizes in the different categories were small, and therefore, we did not fully assess the risk factors and possible multicollinearity between the variables. We recommend carrying out larger studies to assess these factors in the future. Further, this study did not investigate other possible causes of fever that are not detected by conventional bacterial culture methods. We have shown high proportions of ESBL genes, with CTX-M dominating, in Enterobacterales isolated from this patient population [39]. We are pursuing additional genomic studies to further characterize resistance genes in bacterial isolates and to identify nonbacterial pathogens using next-generation sequencing approaches.

In summary, bacteremia was frequent in patients with hematologic cancer and FN and was associated with poor survival at UCI. MDR bacteria were the main cause of bacteremia and were associated with increased mortality. We demonstrate the need for enhanced microbial surveillance and antimicrobial stewardship programs in SSA to identify high-risk patients with cancer for MDR infections and new approaches to prioritize antibiotic utilization strategies. Future studies to understand risk factors associated with the spread of AMR in cancer centers are critical to informing infection prevention and control strategies.

## Supplementary Material

ofae682_Supplementary_Data

## Supplementary Data

Supplementary materials are available at *Open Forum Infectious Diseases* online. Consisting of data provided by the authors to benefit the reader, the posted materials are not copyedited and are the sole responsibility of the authors, so questions or comments should be addressed to the corresponding author.

## Notes

***Acknowledgments.*** The authors acknowledge and thank the Makerere University College of Health Sciences, Uganda Cancer Institute, Fred Hutchinson Cancer Research Center, Hutchison Centre Research Institute Uganda, and Makerere University Clinical Microbiology Laboratory and Genomics and Molecular Biology Laboratory of the Department of Immunology and Molecular Biology for their administrative and technical support. We also thank Prof. Rhoda Morrow and Prof. Anna Wald for their careful review of this manuscript.

***Author contributions.*** All authors contributed to conceptualization, methodology, investigation, formal analysis, original draft preparation, and reviewing and editing. All authors read and approved the final manuscript.

***Financial support.*** This research was funded by the National Institutes of Health (training grant number D43 TW009759 to W. P.); a Uganda Cancer Institute–African Development Bank Training Scholarship (ADM 342/411/01 to M. L.); and an Alliance for Global Health and Science Small Grants award, Center for Emerging and Neglected Diseases, University of California, Berkeley (to M. L.).

***Potential conflicts of interest.*** All authors: No reported conflicts.
