# Supplementary material for: Etiology, Risk Factors, and Outcomes of Bacteremia in Patients With Hematologic Malignancies and Febrile Neutropenia in Uganda
Source: Open Forum Infect Dis. 2024 Nov 16;11(12):ofae682. doi: 10.1093/ofid/ofae682 (PMC11630766; doi:10.1093/ofid/ofae682)
Supplement: ofae682_Supplementary_Data [file ofae682_supplementary_data.zip › Supplemental Tables.docx]

Supplementary Table 1: Patient characteristics by FNE status were analyzed using univariate testing with and without adjusting adult/ped and HIV status

| Patient characteristics | Overall | FNE | No FNE | Univariate | p | Univariate adjust adult/ped and HIV status | P |
| --- | --- | --- | --- | --- | --- | --- | --- |
|  | N | N (%) | N(%) | OR (95% CI) |  | OR (95%) |  |
| Total | 495 | 132 (27) | 363 (73%) |  |  |  |  |
| Age (years, median) | 23 (IQR:11-42) | 19 (10-26) | 25 (11-47) | 0.98 (0.95, 0.99) | <0.01 |  |  |
| Ward |  |  |  |  |  |  |  |
| Adult | 315 | 83 (26) | 232 (74) | Ref |  |  |  |
| Pediatric | 180 | 49 (27) | 131 (73) | 1.05 (0.69, 1.58) | 0.83 |  |  |
| Gender |  |  |  |  |  |  |  |
| Female | 189 | 49 (26) | 140 (74) | Ref |  | Ref |  |
| Male | 306 | 83 (27) | 223 (73) | 1.06 (0.71, 1.61) | 0.77 | 0.95 (0.62, 1.46) | 0.8 |
| Cancer Diagnosis |  |  |  |  |  |  |  |
| ALL | 106 | 42 (40) | 64 (60) | Ref |  | Ref |  |
| AML | 94 | 52 (55) | 42 (45) | 1.89 (1.08, 3.33) | 0.03 | 1.74 (0.97, 3.14) | 0.07 |
| CLL | 10 | 1 (10) | 9 (90) | 0.17 (0.02, 1.39) | 0.10 | 0.13 (0.01, 1.74) | 0.06 |
| CML | 33 | 5 (15) | 28 (85) | 0.27 (0.09, 0.71) | 0.01 | 0.23 (0.07, 0.62) | 0.01 |
| HL | 27 | 3 (11) | 24 (89) | 0.19 (0.04, 0.59) | 0.01 | 0.17 (0.04, 0.52) | 0.01 |
| NHL | 166 | 22 (13) | 144 (87) | 0.23 (0.13, 0.42) | <0.001 | 0.27 (0.14, 0.49) | <0.001 |
| Multiple Myeloma | 39 | 1 (3) | 38 (97) | 0.04 (0. 0.2) | <0.01 | 0.04 (0, 0.18) | <0.01 |
| MDS | 19 | 6 (32) | 13 (68) | 0.7 (0.23, 1.93) | 0.51 | 0.51 (0.16, 1.46) | 0.23 |
| Other | 1 | 0 (0) | 1 (100) | - | - |  |  |
| HIV status |  |  |  |  |  |  |  |
| Positive | 55 | 5 (9) | 50 (91) | Ref |  |  |  |
| Negative | 318 | 111 (35) | 207 (65) | 5.36 (2.28, 15.76) | <0.01 |  |  |
| Unknown | 122 | 16 (13) | 106 (87) | 1.51 (0.56, 4.82) | 0.45 |  |  |
| Previous hospitalization |  |  |  |  |  |  |  |
| No | 216 | 47 (22) | 169 (78) | Ref |  | Ref |  |
| Yes | 279 | 85 (30) | 194 (70) | 1.58 (1.05,2.39) | 0.03 | 1.7 (0.11, 2.62) | 0.02 |

ALL: acute lymphocytic leukemia; AML: acute myeloid leukemia; CLL: chronic lymphocytic leukemia; CML: chronic myeloid leukemia; HL: Hodgkin lymphoma; NHL: Non-Hodgkin lymphoma; MDS: myelodysplastic syndrome; FNE: febrile neutropenia episode

Supplementary Table 2: Epidemiological and clinical characteristics of patients by bacteremia status using univariate testing and a multivariate model “Logit(bacteremia/no bacteremia) ~ Age + Chemo at FNE + ANC<100 + Hypotension + Mucositis”

| Patient characteristics | Overall | Bacteremia | No Bacteremia | Univariate | p | Multivariate | P |
| --- | --- | --- | --- | --- | --- | --- | --- |
|  |  | N | N | OR (95% CI) |  | OR (95% CI) |  |
| Total | 132 | 43 | 89 |  |  |  |  |
| Age (years, median) | 19 | 16 (10-26) | 20 (10-34) | 0.98 (0.95, 1) | 0.05 | 0.99 (0.95, 1) | 0.12 |
| Patient on chemotherapy at FNE |  |  |  |  |  |  |  |
| No | 70 | 17 (24) | 53 (76) | Ref |  | Ref |  |
| Yes | 62 | 26 (42) | 36 (58) | 2.25 (1.08, 4.8) | 0.03 | 2.03 (0.92,4.58) | 0.08 |
| ANC<100 |  |  |  |  |  |  |  |
| No | 81 | 19 (23) | 62 (77) | Ref |  | Ref |  |
| Yes | 51 | 24 (47) | 27 (53) | 2.9 (1.38, 6.23) | 0.01 | 2.04 (0.88,4.71) | 0.09 |
| Hypotension |  |  |  |  |  |  |  |
| No | 104 | 29 (28) | 75 (72) | Ref |  | Ref |  |
| Yes | 28 | 14 (50) | 14 (50) | 2.46 (1.03, 5.93) | 0.04 | 2 (0.76,5.06) | 0.16 |
| Mucositis |  |  |  |  |  |  |  |
| No | 98 | 26 (27) | 72 (73) | Ref |  | Ref |  |
| Yes | 34 | 17 (50) | 17 (50) | 2.77 (1.23, 6.27) | 0.01 | 2.13 (0.87, 5.22) | 0.1 |

FNE: febrile neutropenia episode; ANC: absolute neutrophil count

Supplementary Table 3: Bacteria isolated in the different populations (adults/peds)

| **Bacteria** | **Number** |
| --- | --- |
| **Adults** |  |
| *E. coli* | 15 |
| *K. Pneumoniae* | 10 |
| *Enterobacter sp* | 1 |
| *Citrobacter sp* | 1 |
| *P. aeruginosa* | 2 |
| *Enterococcus sp* | 4 |
| *S. aureus* | 2 |
| Coagulase negative Staphylococcus | 1 |
| *Streptococcus oralis* | 2 |
| *Viridans Streptococcus* | 1 |
| **Total** | **39** |
|  |  |
| **Peds** |  |
| *E. coli* | 10 |
| *K. Pneumoniae* | 6 |
| *Enterobacter sp* | 1 |
| *Leclerica adecarboxylata* | 2 |
| *Salmonella sp* | 1 |
| *Empedobacter brevis* | 1 |
| *Enterococcus sp* | 3 |
| *Streptococcus sp* | 1 |
| **Total** | **25** |

Supplementary Table 4: Polymicrobial bloodstream infections in patients with hematologic malignancies and febrile neutropenia

| Cancer type | Ward | HIV Status | poly | Organisms | Org |
| --- | --- | --- | --- | --- | --- |
| AML | pediatric | Negative | gp, gn | *E. coli, E. coli, E. coli, Enterococcus sp* | 4 |
| ALL | pediatric | Negative | gn, gn | *K. pneumoniae, K. pneumoniae* | 2 |
| AML | pediatric | Unknown | gn, ot | *Salmonella sp, mold* | 2 |
| AML | pediatric | Negative | gn, gn | *Enterobacter spp, Leclerica adecarboxylata, Leclerica adecarboxylata* | 3 |
| CML | adult | Negative | gp, gp | *S. aureus, CNS* | 2 |
| HL | pediatric | Negative | gn, ot | *K. pneumoniae, mold* | 2 |
| AML | adult | Negative | gp, gn | *E. coli, Enterococcus sp* | 2 |
| ALL | adult | Negative | gn, gn | *E. coli, E. coli* | 2 |
| AML | adult | Negative | gn, gn | *E. coli, E. coli, K. pneumoniae* | 3 |
| MDS | adult | Negative | gn, gn | *E. coli, P. aeruginosa* | 2 |
| AML | adult | Negative | gn, gn | *E. coli, E. coli, K. pneumoniae* | 3 |
| ALL | adult | Negative | gp, gn | *E. coli, E. coli, Enterococcus sp, Streptococcus oralis* | 4 |
| ALL | adult | Negative | gn, gn | *E. coli, K. pneumoniae* | 2 |
| ALL | adult | Negative | gp, gn | *E. coli, Enterococcus sp* | 2 |
| AML | adult | Negative | gn, gn | *E. coli, E. coli, K. pneumoniae* | 3 |

Supplementary Table 5: Characteristics of patients with hematologic cancers and bacteremia who were dead at 30 days from febrile neutropenia

| ped/adult | cancer type | HIV status | Vital stautus | organism(s) | app/inapp 48h |
| --- | --- | --- | --- | --- | --- |
| ped | AML | Negative | Dead | *E. coli, E. coli, E. coli, Enterococcus sp* | No |
| ped | AML | Negative | Dead | *K. pneumoniae* | No |
| adult | AML | Negative | Dead | *Enterococcus sp* | No |
| ped | ALL | Negative | Dead | *Enterococcus sp* | No |
| ped | ALL | Negative | Dead | *K. pneumoniae, K. pneumoniae* | No |
| ped | ALL | Negative | Dead | *Enterococcus sp* | No |
| ped | AML | Unknown | Dead | *Salmonella sp,* mold | No |
| ped | ALL | Negative | Dead | *K. pneumoniae* | Yes |
| ped | AML | Negative | Dead | *E. coli* | No |
| adult | AML | Negative | Dead | *E. coli, Enterococcus sp* | No |
| adult | ALL | Negative | Dead | *E. coli, E. coli* | No |
| adult | AML | Negative | Dead | *E. coli, E. coli, K. pneumoniae* | No |
| adult | MDS | Negative | Dead | *E. coli, P. aeruginosa* | No |
| adult | AML | Negative | Dead | *E. coli, E. coli, K. pneumoniae* | No |
| adult | ALL | Negative | Dead | *E. coli, E. coli, Enterococcus sp, Streptococcus oralis* | No |
| adult | ALL | Negative | Dead | *K. pneumoniae* | No |
| adult | ALL | Negative | Dead | *E. coli, Enterococcus sp* | No |
| adult | AML | Negative | Dead | *E. coli* | No |
| ped | Other | Negative | Dead | *E. coli* | No |
| ped | HL | Negative | Dead | *Citrobacter sp* | No |
| adult | ALL | Negative | Dead | *K. pneumoniae* | Yes |
| adult | NHL | Negative | Dead | *K. pneumoniae* | Yes |

app: appropriate; inapp: inappropriate

Supplementary Figure 1: Prospective cohort of hematologic cancer patients

Supplemental Figure 2: Documented antibiotics in all 132 episodes of febrile neutropenia. Of the 132 febrile episodes, at least one antibiotic was documented for 65 participants with febrile neutropenia. More than one antibiotic was documented for 21 participants. The most common combination included piperacillin-tazobactam and gentamicin.
