# Supplementary figures and images for: Etiology, Risk Factors, and Outcomes of Bacteremia in Patients With Hematologic Malignancies and Febrile Neutropenia in Uganda
Source: Open Forum Infect Dis. 2024 Nov 16;11(12):ofae682. doi: 10.1093/ofid/ofae682 (PMC11630766; doi:10.1093/ofid/ofae682)

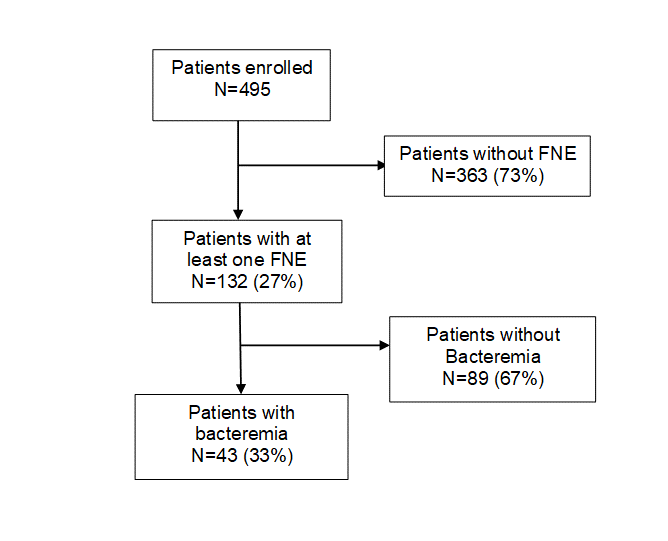

Supplement: ofae682_Supplementary_Data [file ofae682_supplementary_data.zip › Supp. Fig 1.tif]

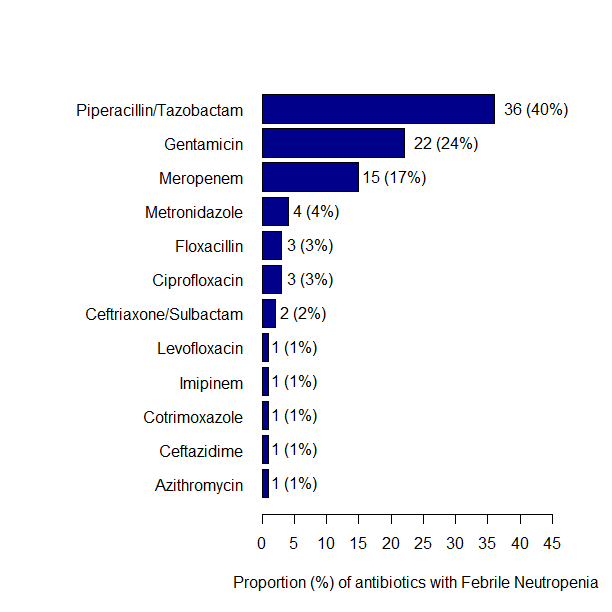

Supplement: ofae682_Supplementary_Data [file ofae682_supplementary_data.zip › Supp. Fig 2.tiff]
